# Prolonged resuscitation of metabolic acidosis after trauma is associated with more complications

**DOI:** 10.1186/s13018-015-0288-3

**Published:** 2015-09-24

**Authors:** Douglas S. Weinberg, Arvind S. Narayanan, Timothy A. Moore, Heather A. Vallier

**Affiliations:** Department of Orthopaedic Surgery, MetroHealth Medical Center, Case Western Reserve University, 2500 MetroHealth Dr., Cleveland, OH 44109 USA; Case Western Reserve University, School of Medicine, 10900 Euclid Avenue, Cleveland, OH 44106 USA

**Keywords:** Early appropriate care, Acidosis, Fixation timing, Resuscitation, Polytrauma

## Abstract

**Background:**

Optimal patterns for fluid management are controversial in the resuscitation of major trauma. Similarly, appropriate surgical timing is often unclear in orthopedic polytrauma. Early appropriate care (EAC) has recently been introduced as an objective model to determine readiness for surgery based on the resuscitation of metabolic acidosis. EAC is an objective treatment algorithm that recommends fracture fixation within 36 h when either lactate <4.0 mmol/L, pH ≥ 7.25, or base excess (BE) ≥−5.5 mmol/L. The aim of this study is to better characterize the relationship between post-operative complications and the time required for resuscitation of metabolic acidosis using EAC.

**Methods:**

At an adult level 1 trauma center, 332 patients with major trauma (Injury Severity Score (ISS) ≥16) were prospectively treated with EAC. The time from injury to EAC resuscitation was determined in all patients. Age, race, gender, ISS, American Society of Anesthesiologists score (ASA), body mass index (BMI), outside hospital transfer status, number of fractures, and the specific fractures were also reviewed. Complications in the 6-month post-operative period were adjudicated by an independent multidisciplinary committee of trauma physicians and included infection, sepsis, pulmonary embolism, deep venous thrombosis, renal failure, multiorgan failure, pneumonia, and acute respiratory distress syndrome. Univariate analysis and binomial logistic regression analysis were used to compare complications between groups.

**Results:**

Sixty-six patients developed complications, which was less than a historical cohort of 1,441 patients (19.9 % vs. 22.1 %). ISS (*p* < 0.0005) and time to EAC resuscitation (*p* = 0.041) were independent predictors of complication rate. A 2.7-h increase in time to resuscitation had odds for sustaining a complication equivalent to a 1-unit increase on the ISS.

**Conclusions:**

EAC guidelines were safe, effective, and practically implemented in a level 1 trauma center. During the resuscitation course, increased exposure to acidosis was associated with a higher complication rate. Identifying the innate differences in the response, regulation, and resolution of acidosis in these critically injured patients is an important area for trauma research.

**Level of evidence:**

Level 1: prognostic study.

## Background

The timing of osteosynthesis in multiple-injured patients is controversial. Many authors have promoted damage control orthopedics (DCO), an initially conservative approach for severely injured patients as a means to decrease post-operative complications [1–6]. However, this comes at the expense of providing early definitive fracture care, which improves posture, accelerates ambulation, and reduces complications related to inactivity [7–15]. While early definitive fixation and DCO both offer undeniable benefits in certain clinical situations, establishing appropriate patient criterion remains elusive.

Recently, early appropriate care (EAC) was introduced as an objective algorithm to determine surgical readiness. EAC quantifies the resuscitation of metabolic acidosis to enhance patient selection between early total care and DCO. Statistical modeling showed that complications are decreased when definitive fixation occurred within 36 h of injury with at least one of the following: lactate <4.0 mmol/L, pH ≥7.25, or base excess (BE) ≥−5.5 mmol/L. This protocol offers the additional advantages of being simple, easily applicable to every trauma patient, and has been designed and reviewed by a multidisciplinary team of trauma providers [16].

However, the simplicity afforded by the EAC guidelines does not incorporate timing over which the resuscitation of acidosis occurs. With the abundance of recent literature emphasizing the importance of optimal fluid management, it would therefore be clinically prudent to determine if the timing to resuscitation in patients managed with EAC affected post-operative complication rate [17–20]. Consequently, we designed a prospective study to investigate the relationship between the EAC resuscitation course and the development of post-operative complications.

## Methods

### Implementing early appropriate care at a level 1 trauma center

Following institutional review board (MetroHealth Institutional Review Board) approval, patients were prospectively managed with early appropriate care from October 2010 through March 2013 at a level 1 adult trauma center. EAC treatment guidelines recommended definitive fixation of the orthopedic fractures within 36 h of injury once resuscitation of initial acidosis had improved such that lactate <4.0 mmol/L, pH ≥7.25, or BE ≥−5.5 mmol/L [16]. Definitive fixation was defined as an orthopedic intervention was intended to represent final treatment.

Patients were excluded for having injuries sustained from low-energy mechanisms (252), being skeletally immature (23), or having an Injury Severity Score (ISS) <16 (190). Patients with definitive nonoperative treatment were also excluded (835). Patients who received definitive orthopedic fixation before being appropriately resuscitated were excluded (three). One thousand one hundred and seventy-eight patients were excluded in total, and three hundred and thirty-two adult patients were enrolled.

### Timing to resuscitation

The time of injury was available in all 332 patients enrolled. A lactate value was ordered on all patients in the initial trauma workup, and arterial blood gas analysis was obtained at the discretion of the attending general trauma surgeon. Lab values were repeated in 8-h increments until they had normalized. All patients were off presser support at the time of surgery. The time from injury to EAC protocol resuscitation was recorded. Other data obtained for all patients included age, race, gender, ISS, American Society of Anesthesiologists score (ASA), body mass index (BMI), outside hospital transfer status, types of fractures, and types and severity of injury to other systems.

### Measurements of outcome

Complications in the 6-month post-operative period were determined by an independent adjudication committee that was not involved in the development of the EAC protocol or any of its earlier research. The committee consisted of physicians from a multidisciplinary background, including those specializing in general trauma surgery/critical care, orthopedic surgery, neurosurgery, and anesthesiology. Patients were assessed for acute renal failure [21], multiple organ failure [22], sepsis [23], deep venous thrombosis (DVT) [21], infection, and pulmonary complications: pneumonia [24], acute respiratory distress syndrome (ARDS) [25], and pulmonary embolism (PE) [26].

### Statistical analysis

Multiple regression analysis was performed to determine the independent predictors of time required to EAC protocol resuscitation. All univariate data was analyzed for skewness and kurtosis. Overall complication rate was compared to a historical cohort. Comparisons between groups were performed with the independent samples Mann-Whitney *U* test, or independent samples *t* test for continuous variables, where parametrically appropriate. The Fisher’s exact test was used for categorical variables. Stepwise binomial logistic regression with backward selection was performed to identify independent clinical predictors of complication rate, with the same group of comparisons that were performed for the univariate analysis. Variables with a *p* value of less than 0.20 in univariate comparison were chosen as candidates for the binomial logistic model, with significance determined with use of the likelihood ratio chi-square test. Significance was set at a *p* value of less than 0.05 in regression testing. Adjusted odds ratios and 95 % confidence intervals were derived. Receiver operating curves were generated. Statistical analysis was performed with the SPSS (version 22.0; IBM Incorporated, Armonk, NY) software package. Significance was set at *p* ≤ 0.05.

## Results

### Descriptive data

Three hundred and thirty-two patients were prospectively assessed and treated according to EAC criteria, including 96 females (28.9 %) and 236 males with an average age of 39 ± 16 years. Mean BMI was 29.6 ± 7.8, and mean ISS was 27 ± 12. There were a total of 376 fractures in these patients (1.14 fractures/patient) with 171 fractures of the femur, 56 fractures of the acetabulum, 70 fractures of the pelvic ring, six fractures of the cervical spine, and 73 fractures of the thoracolumbar spine. The mean time required to achieve EAC resuscitation goal was 6.8 ± 7.8 h (range, 0.3 to 35.3). Sixty-six patients sustained at least one post-operative complication (19.9 %); while 266 did not have any complications, this was slightly less than a historical control of 1443 patients with a 22.1 % complication rate (*p* = 0.155).

Transfer from an outside hospital was an independent predictor of more time needed to achieve EAC resuscitation, requiring an additional 1 h and 10 min of resuscitation time on average (standardized beta 0.112, *p* = 0.045). The time required for EAC resuscitation was independent of age, race, gender, ISS, ASA, BMI, number of fractures, and any specific fractures (Table 1). All correlations in the multiple regression analysis were below 0.5. All VIF values were below 10 and coefficient tolerances above 0.10. Normal probability plots of the regression standardized residual were inspected for normality. Scatterplots of the residuals were inspected for homoscedasticity, and a Cook’s distance <1 confirmed any undue influence from outliers.

**Table 1:** Multiple regression results, independent predictors of time to EAC resuscitation

Independent variable	Standardized beta^a^	Significance
Age	0.030	0.596
African-American race	0.070	0.511
Gender	−0.005	0.935
Injury Severity Score (ISS)	−0.005	0.935
American Society of Anesthesiologists score (ASA)	−0.039	0.578
Body mass index (BMI)	0.018	0.753
Transfer from outside hospital (OSH)	0.112	0.045†
Number of fractures	−0.111	0.296
Presence of a femur fracture	−0.036	0.735
Presence of an acetabular fracture	0.029	0.736
Presence of a pelvic ring fracture	0.075	0.403
Presence of a cervical spine fracture	0.096	0.089
Presence of a thoracolumbar spine fracture	0.065	0.712

†*p* < 0.05

^a^A positive value for race suggests that African-Americans took longer to receive resuscitation. A positive value for gender suggests that females took longer to receive resuscitation. A positive value for transfer from outside hospital indicates that outside transfer took longer to receive resuscitation

### Univariate analysis

The 66 patients who developed a post-operative complication had higher ISS scores (*p* < 0.0005), higher ASA scores (*p* < 0.0005), significantly increased time to EAC resuscitation (*p* = 0.022, and were more likely to have pelvic ring (*p* = 0.027), and thoracolumbar spine (*p* = 0.045) fractures. Patients with femur fractures had fewer complications than those with other fractures (*p* = 0.005), and female patients showed a trend towards decreased complications (*p* = 0.070) (Table 2).

**Table 2:** Univariate analysis for patients with post-operative complications

Variable	No complication^a^	Complication^a^	Significance
	*n* = 266 (80.1 %)	*n* = 66 (19.9 %)	
Age (years)	39.1 ± 16.2	40.3 ± 17.3	0.597
African-American race	53 (19.7 %)	15 (22.7 %)	0.505
Female gender	82 (31.1 %)	13 (19.7 %)	0.070
ISS	24.5 ± 9.3	36.4 ± 15.0	<0.0005†
ASA score	2.7 ± 0.8	3.3 ± 0.9	0.020†
BMI	29.5 ± 7.9	30.3 ± 7.7	0.483
OSH transfer	113 (42.5 %)	30 (45.5 %)	0.679
Time to EAC resuscitation (hours)	6.29 ± 8.24	9.07 ± 10.58	0.022†
Number of fractures^b^	1.1 ± 0.4	1.2 ± 0.4	0.331
Presence of a femur fracture^c^	138 (51.9 %)	21 (31.8 %)	0.005†
Presence of an acetabulum fracture	46 (17.3 %)	10 (15.2 %)	0.854
Presence of a pelvic ring fracture	49 (18.4 %)	21 (31.8 %)	0.027†
Presence of a cervical spine fracture	3 (1.1 %)	3 (4.5 %)	0.096
Presence of a thoracolumbar spine fracture	52 (19.5 %)	21 (31.8 %)	0.045†

*ISS* Injury Severity Score, *ASA* American Society of Anesthesiologists’ Classification, *BMI* body mass index, *OSH* outside hospital transfer

†*p* < 0.05

^a^The values are given as the mean and the standard deviation for continuous variables and as the number of patients in the respective outcome group, with the percentage in parentheses for categorical variables

^b^Compared with the chi-square test

^c^Twelve patients sustained bilateral femur fractures. Since the univariate analysis was designed to assess the presence or absence of a femur fracture, the number of individuals with femur fractures (159) is less than and discordant with the number of femur fractures in total (171)

### Multivariate analysis

Four independent predictors of complications were identified (Table 3). Complications were fewer in females (odds ratio [OR] = 0.428, 95 % confidence interval [CI] 0.202–0.908, *p* = 0.027). Every 1-point increase on the ISS scale was associated with a 8.7 % increase in the odds of sustaining a complication ([OR] = 1.087, [CI] 1.061–1.118, *p* < 0.0005). For each 1-h increase in the time required to reach EAC resuscitation, there was a 3.2 % increase in the odds of sustaining a complication ([OR] = 1.032, [CI] 1.001–1.063, *p* = 0.041). The presence of a femur fracture was a negative predictor of complications ([OR] = 0.447, [CI] 0.236–0.845, *p* = 0.013).

**Table 3:** Multivariate analysis, binomial logistic regression results, independent predictors of complications

Independent variable	Regression coefficient (B)	Wald statistic	Significance	Odds ratio	95 % CI for odds ratio	
					Lower	Upper
Female gender	−0.848^a^	4.896	0.027†	0.428	0.202	0.908
ISS	0.084	43.957	<0.0005†	1.087	1.061	1.115
Time to resuscitation	0.031	4.180	0.041†	1.032	1.001	1.063
Presence of femur fracture	−0.806^b^	6.150	0.013†	0.447	0.236	0.845
Constant	−2.696	19.090	<0.0005	0.067		

A 2.7-h increase in time to resuscitation had equivalent odds for sustaining a complication as a 1-unit increase on the ISS

*ISS* Injury Severity Score

†*p* < 0.05

^a^A negative value for gender suggests females suffer fewer complications than males

^b^A negative value for femur fracture suggests that patients with a femur fracture suffer fewer complications than those without

ASA score, the presence of a pelvic ring injury, and the presence of a thoracolumbar spine fracture were not independent predictors of sustaining a complication. The logistic regression model was statistically significant, *χ*^2^(4) = 64.718, *p* < 0.0005. All correlations were below 0.5. All VIF values were below 10 and coefficient tolerances above 0.10. Scatterplots of the residuals were inspected for homoscedasticity, and a Cook’s distance <1 confirmed any undue influence from outliers.

### Cutoff points for time resuscitation

**Fig. 1:**
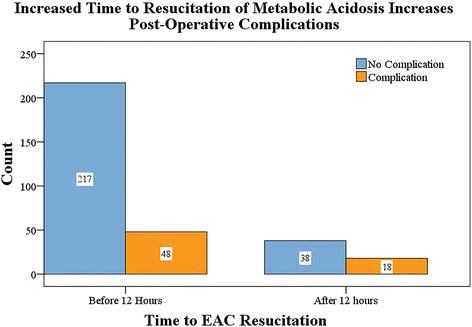
Number of complications based on time to EAC resuscitation

In the continued design and practical application of EAC, it may be advantageous to categorize timing of resuscitation into cutoff hours. The different cutoff points extrapolated from the receiver operating curves suggested that there was no clear time during EAC resuscitation after which point the risk of having a complication increased markedly (Table 4). This confirmed the linear relationship between timing to EAC resuscitation and complication risk. A cutoff value of 12 h was chosen to plot the values in Fig. 1.

**Table 4:** Sensitivity, specificity, PPV, NPV, and accuracy for predicting a complication given specific cutoff times during resuscitation course

Cutoff time (hours)	Sensitivity (%)	Specificity (%)	PPV (%)	NPV (%)	Accuracy (%)
2	74	26	50	65	50
3	70	36	52	54	53
6	46	67	57	55	56
9	35	82	66	56	58
11.25	32	86	70	56	61
12	26	87	66	54	59
18	12	91	67	51	52
24	9	96	69	51	52

*PPV* positive predictive value, *NPV* negative predictive value

## Discussion

Resuscitation is of paramount importance in the management of trauma patients. Historically, this proceeded with intravenous fluid with a principal goal of normalizing hemodynamic status [17, 27–29]. However, critical care providers later began to focus on the poor outcomes in patients who became coagulopathic (INR >1.5), hypothermic (temperature <35 °C), and acidotic (pH <7.2). Rotondo and Zonies described this as the “trauma triad of death” in 1997; this has since prompted trauma systems to investigate a number of different approaches for managing fluid resuscitation [28].

Recent literature has recommended that trauma providers adopt a “less is more” approach in attempts to decrease complications [30]. The American College of Surgeons’ Advanced Trauma Life Support (ATLS) guidelines recommend isotonic fluid administration during the resuscitation of adult polytrauma [31]. As Feinman et al. remind us, this only transiently increases intravascular volume: one third of isotonic fluid remains extracellular—of which, only one fourth remains intravascular. The remaining fluid becomes interstitial, with profound consequences on inflammation, microvascular compliance, and cell volume regulatory mechanisms. The resulting tissue edema can provoke acute coronary syndrome, respiratory compromise, and end-organ failure, among other devastating complications [17].

It is therefore not surprising that considerable effort has been devoted to establishing appropriate endpoints for trauma resuscitation. EAC was spawned with this goal in mind: creating an algorithm for the resuscitation of severe orthopedic polytrauma that optimizes surgical timing for fractures of the femur, acetabulum, pelvic ring, and spine [16]. Earlier reports have shown decreased complications compared to historical cohorts. The protocol recommends definitive stabilization within 36 h if at least one of the following three conditions is met: lactate <4.0 mmol/L, pH ≥7.25, or BE ≥−5.5 mmol/L. EAC is simple and convenient, and preliminary results suggest that it is safe and effective [32]. However, to our knowledge, there exists limited data regarding the impact of resuscitative delay on post-operative complications using the EAC—or any alternative—resuscitative biomarkers.

In this prospective analysis, the only independent predictor of delay in resuscitation was transfer from an outside hospital. This result is not surprising, as transfer of care in any trauma system inherently introduces delays. Furthermore, there has been a recent trend towards delaying fluid administration for these patients, for many of the reasons discussed above [33]. One may have expected the more critically injured patients to have longer resuscitation courses; however, this was not the case in this cohort or others conducted previously [34].

Predictably, these more critically injured patients had more complications. Tornetta et al. and other authors have suggested that complications in multiple-injured patients with fracture are most strongly associated with the nature of the pre-hospital condition [35]. Our results showed that delays in resuscitation were directly related to complication rate. The most obvious explanation would assume that these patients were more severely injured and required a more complete resuscitation course that resulted in surgical delay—all factors that have been linked to poor outcomes. However, this was not the case: ISS was not a significant predictor of resuscitation time, and moreover, the endpoint of resuscitation was an independent predictor of complication rate.

It is more likely that these complications were direct consequences of prolonged acidosis. In severe polytrauma, acid-base homeostasis is disrupted by inflammatory cytokines. Even with judicious allocation of bicarbonate reserve, the amount available may be insufficient for the compromised homeostatic mechanisms to mount an effective response [36]. These severely injured patients with impaired acid-base regulation and prolonged exposure to acidotic environment were then not surprisingly at an increased risk for complications and, in other studies, mortality [37]. Regression modeling confirmed a direct linear relationship between time to EAC protocol resuscitation and complication rate, and while a time point of slightly under 12 h of resuscitation maximized accuracy of this model, there existed no clear inflection point to suggest establishing a firm cutoff goal for completion of resuscitation. Females were also shown to have decreased complications, perhaps for reasons related to improved response to acid-base perturbations. Previous authors have demonstrated femoral shaft fractures to have lower complication rates than other fractures, perhaps given the comparatively short surgical duration and relatively little associated surgical blood loss [38].

Morshed et al. reviewed a large database of multiple-injured patients with femur fractures and showed that complications were minimized when fixation was performed between 12–24 h from the time of injury. Since surgical criteria vary widely between trauma networks, the authors inferred that early fracture fixation was a direct surrogate for under-resuscitation. They therefore concluded that incomplete resuscitation was a likely cause of complications [39]. Similarly, while our results show a significant correlation between the time to resuscitation and complications in polytrauma, this study does not establish causation. Cause and effect remain unclear. Moreover, the effects of delayed resuscitation were modest at best compared to injury severity, with a 2.7-h delay in resuscitation having equivalent odds for sustaining a complication as 1-unit increase on the ISS. This serves as another humbling reminder that although interventions performed in the arena of trauma care are crucial, frequently, patient outcomes are very strongly determined by the nature of their pre-hospital condition. However, as Morshed et al. remind us, fracture fixation represents an essential part of the resuscitation process, especially for certain fractures of the pelvic ring and femur that may be associated with massive hemorrhage.

Going forward, it would be imprudent to advise expedited resuscitation based on this data alone. This experiment made no attempt at quantifying how (or at what rate) EAC resuscitation was to be achieved. In doing so, we were only able to monitor the effective “outputs” of this resuscitation, with little information regarding the “inputs” of treatment. These decisions were made by the general trauma surgery team, who incorporated many of the complicated considerations discussed earlier.

Nonetheless, there clearly exist important differences among individuals in the response to insults of the acid-base system. Identifying these organic differences, and perhaps manipulating them to optimize resuscitation, remains elusive and an important area for future research. This should be especially relevant for patients with existing medical conditions such as diabetes, renal failure, and chronic obstructive pulmonary disease, all of which have impaired regulation of acid-base balance [40]. Others with ischemic heart disease or congestive heart failure may also have physiological limitations making a slightly longer resuscitative course safer and more desirable. This study, and the EAC criterion as a whole, do not account for these or any other medical comorbidities. It would therefore be important to determine which of these conditions were independent predictors of complications and then thoroughly analyze their resuscitation course. This represents an essential area for continued study.

Other important limitations of EAC are that it is exclusively dependent on metabolic acidosis to determine surgical timing. However, the design of EAC affords consideration of multiple lab values, allowing one to choose biomarkers based on institution-specific order panels, practitioner familiarity, or in consolidation with tests ordered for other purposes. While each of the three resuscitative parameters used are expected to be collinear with one another, this allows versatility in implementation. However, its statistical design may incompletely characterize the relationship between specific injury patterns and biomarkers. While the nature of any universal protocol is appealing to trauma providers, it may be necessary to adjust cutoff parameters based on anticipated blood loss and surgical duration. This study was designed to investigate definitive treatment patterns and did not incorporate the timing of skeletal traction or any other external fixation device; this has been extensively reported previously. Other limitations of EAC have been discussed previously [32]. Similarly, providers should consider other aspects of patient, surgeon, and hospital readiness before surgery. Strengths of the study include a large patient sample size with prospectively collected data, consistencies in patient care, with standardized methods for DVT prophylaxis, perioperative antibiotics, ventilator management, and nutrition. In addition, all patients met the resuscitation criteria within 36 h.

## Conclusion

In summary, resuscitation with EAC criteria has been shown to decrease complications compared to historical cohorts. Definitive osteosynthesis should proceed when one of the following parameters is adequately resuscitated: lactate <4.0 mmol/L, pH ≥7.25, or BE ≥−5.5 mmol/L. However, we would recommend that readers rely on their clinical experience to make these decisions based on their physical examination, other laboratory findings, and any operational issues related to their specific trauma system. During the course of resuscitation, prolonged exposure to acidosis is associated with a greater risk for complications. Further characterizing the relationships between acidosis, resuscitation course, and complications are required. Early reports such as this spark the need for future study in more patients across multiple institutions.

## Consent

Written informed consent was obtained from the patient for the publication of this report and any accompanying images.

## Abbreviations

DCO: damage control orthopedics
EAC: early appropriate care
